# Identifying protons trapped in hematite photoanodes through structure–property analysis†

**DOI:** 10.1039/c9sc04853g

**Published:** 2019-12-16

**Authors:** Yutong Liu, Rodney D. L. Smith

**Affiliations:** Department of Chemistry, University of Waterloo 200 University Avenue W. Waterloo Ontario N2L 3G1 Canada rodsmith@uwaterloo.ca; Waterloo Institute for Nanotechnology, University of Waterloo 200 University Avenue W. Waterloo Ontario N2L 3G1 Canada

## Abstract

Uncertainty regarding the nature of structural defects in hematite and their specific impacts on material properties and photoelectrocatalytic water oxidation inhibits their development as photoanodes. We perform structure–property analysis on a series of hematite films fabricated by annealing lepidocrocite films with varied temperatures, annealing times and atmospheres and find a gradient in the magnitude of a crystal lattice distortion by tracking the relative intensity of a formally Raman inactive vibrational mode. Structure–property analysis reveals that this feature in the Raman spectrum correlates to photocurrent density, semiconductor band positions, and the onset of photoelectrocatalysis. We propose that the onset of photoelectrocatalysis is linked to the location of defects that act as intraband recombination sites; an increase in the degree of structural distortion shifts these states towards the conduction band, thereby facilitating recombination. Analysis of the nature of the key Raman vibrations, X-ray diffraction patterns, and the synthetic conditions leads us to assign the distortion to iron vacancies that are induced by the trapping of protons within the crystal lattice. The ability to rapidly diagnose a specific structural defect will aid in the optimization of fabrication protocols for hematite photoanodes.

## Abstract 2

Features in Raman spectra are found to correlate to the band structure and photoelectrochemical behavior across a series of hematite photoanodes. Analysis suggests the presence of iron vacancies caused by the trapping of interstitial protons.

## Graphical abstract

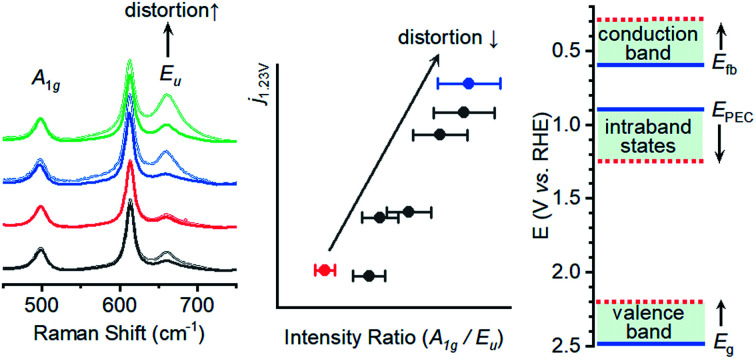

## Introduction

Photoelectrochemical (PEC) reactions are widely viewed as a viable means to harvest solar energy and store it in the form of chemical bonds.^1^ The most prominent approach in such solar fuels research views the oxygen evolution reaction (OER) as a supply of electrons and protons for fuel-forming cathodic reactions such as the hydrogen evolution reaction or electrochemical CO_2_ reduction.^2^ The critical role of OER in solar fuels has led to extensive research and development on manipulating the composition of photoanodes for OER, on tuning fabrication protocols, on interface design, on photophysical analyses, and on controlling nanostructure and morphology.^3–11^ Of the numerous semiconductors that are viable photoanodes for OER,^12^ hematite (α-Fe_2_O_3_) remains one of the most attractive materials. Composed of an earth abundant first-row transition metal, α-Fe_2_O_3_ boasts a near-optimum band gap of *ca.* 2.1 eV and a maximum theoretical photocurrent of 12.6 mA cm^−2^.^13^ The PEC performance records for α-Fe_2_O_3_ photoanodes, however, remain far below these theoretical values. Surface states, defects, or electronic trap states in the crystal lattice are often cited as the reason.

An understanding of the specific defects and their influence on photoelectrochemical behavior is critical to understanding and improving PEC performance. The performance metrics and fundamental physical properties reported for nominally α-Fe_2_O_3_ films are persistently variable across the literature. Examples of variability include properties such as the optical band gap, commonly reported between 1.9 to 2.2 eV,^7,14–17^ the location of the conduction band, which ranges from 0.3 to 0.6 V *vs.* RHE,^7,16,17^ and performance parameters such as the current density at 1.23 V, which varies between *ca.* 1 μA cm^−2^ to 3 mA cm^−2^. The conclusion must thus be drawn that synthetic α-Fe_2_O_3_ films are rarely identical. Techniques developed to identify and locate intraband states^18^ and to probe associated photophysical processes^19^ provide powerful tools to guide development, but without a semblance of consistency across the literature it is difficult to judge exactly how meaningful any single dataset is. Confident identification of specific structural anomalies or defects responsible for variation in properties, and an understanding of how they influence these properties, is an important component in advancing the field.

Efforts to improve α-Fe_2_O_3_ photoanodes have identified oxygen vacancies as a specific structural defect of interest and motivated efforts to gain experimental control over the concentration of oxygen vacancies in α-Fe_2_O_3_.^1^ It has been reported that the concentration of oxygen vacancies (and the associated Fe^II^ sites) can be increased in pure or doped forms of α-Fe_2_O_3_ by annealing under O_2_-deficient environments or decreased by annealing under O_2_-rich environments or in an oxygen plasma.^20–24^ X-ray photoelectron spectroscopy and X-ray absorption spectroscopy have been successfully employed to confirm and quantify these vacancies,^20,21^ and to show that complete removal of oxygen vacancies from the photoanode surface can result in suppression of PEC performance.^20,21^ Increased concentrations have been shown to increase both the density and mobility of charge carriers by orders of magnitude,^25^ a feature which is frequently observed in PEC studies as an increased charge carrier density (*N*_d_) measured through Mott–Schottky analysis;^22–24^ increased *N*_d_ has been widely shown to result in improved PEC performance but induce negligible changes in absorption profiles.^24^ Evidence has been provided that excessive removal of oxygen from the lattice leads to decomposition to Fe_3_O_4_,^25^ suggesting a limitation in potential gains. The improved fundamental understanding of the relationship between PEC behavior and a specific structural defect marks a significant advance that brings the field closer to the rational development of photoanodes. It must be noted, however, that performance metrics for α-Fe_2_O_3_ photoanodes annealed under air or pure oxygen atmospheres have been reported that meet or exceed the performance generally acquired through oxygen vacancy engineering.^26,27^ The picture is thus not complete and there is continued need to establish correlations between fabrication conditions, structural properties and performance parameters.

Herein, we employ structure–property analysis to obtain insight into the specific chemical nature of one such structural defect. We fabricate hematite photoanodes capable of driving OER between 1 μA cm^−2^ and 0.48 mA cm^−2^ at 1.23 V_RHE_ by annealing electrodeposited lepidocrocite (γ-FeOOH) films with varied protocols. The samples exhibit negligible differences in X-ray diffraction patterns but all samples exhibit a formally Raman-inactive absorbance band that signifies a crystal lattice defect. The intensity of the peak for this vibrational mode varies with fabrication protocols and is found to correlate with the band structure descriptors and overall PEC performance. Analysis of the results leads us to propose that the trapping of protons from the γ-FeOOH precursor induces iron deficiency in the lattice that is responsible for the observed behavior.

## Experimental

### Lepidocrocite film deposition

Thin films of lepidocrocite (γ-FeOOH) were electrodeposited on fluorine-doped tin oxide coated glass (FTO) substrates using a literature procedure under neutral conditions.^28^ Samples annealed at 600 °C and below, and those heated at 800 °C for 10 minutes, were deposited on TEC-7 grade FTO (Hartford Glass) while FTO using aluminum borosilicate glass (Solaronix S.A.) was used for samples annealed at 800 °C for 2 hours. All FTO substrates were cleaned by sequential ultrasonication in a detergent solution, Milli-Q H_2_O (18.2 MΩ), and then isopropanol. The substrates were dried under a stream of N_2_ and placed in UV-irradiation chamber (GHO18T5VH lamp, Atlantic Ultraviolet) for 15 minutes. An electrodeposition solution containing 0.02 M ferrous ammonium sulfate hexahydrate (ACS reagent grade, Fisher Chemicals) and 3 M ammonium chloride (ACS reagent grade, EMD Chemicals Inc.) was deaerated with N_2_ for 30 min and then adjusted to pH 7.5 by addition of 0.1 M KOH solution (>85%, Sigma-Aldrich). Application of a constant 0.0 V *vs.* Ag/AgCl (saturated) to FTO substrates submerged in this solution for 7 min at room temperature yielded γ-FeOOH films.

### Lepidocrocite powder fabrication

Fabrication of γ-FeOOH in powder form was accomplished by oxidation of Fe(NH_4_)_2_(SO_4_)_2_·6H_2_O in air.^29^ A 200 mL aliquot of Milli-Q H_2_O in a round bottom flask was acidified by addition of a few drops of 1 M H_2_SO_4(aq)_ (pH 2.4). The solution was purged with N_2_ in an ice bath and a 10.0 g portion of Fe(NH_4_)_2_(SO_4_)_2_·6H_2_O was then dissolved in the flask. Air was bubbled into the solution while continually adding 1 M KOH to maintain a solution pH of 6.5. After 90 minutes the pH of the yellow suspension stabilized at 6.94. The temperature was then increased to 45 °C and bubbled with air. The orange precipitate was collected by vacuum filtration and thoroughly rinsed with Milli-Q H_2_O then dried in an oven at 105 °C.

### Hematite fabrication

A series of α-Fe_2_O_3_ powders and films were prepared by heating the γ-FeOOH samples in a tube furnace. Two temperatures chosen, 600 and 800 °C, match those commonly utilized in the field.^26,28,30,31^ Four different gaseous atmospheres (N_2_, O_2_, N_2_ + H_2_O, O_2_ + H_2_O) were employed to probe the role of oxidizing atmosphere and H_2_O on phase transitions. Humidification of gas streams was accomplished by bubbling the selected purge gas through H_2_O at room temperature immediately before entering the tube furnace. Each of the 8 combinations of temperature and atmosphere was executed with a 2 hour heating period and an additional set of 4 samples was created by heating at 800 °C for 10 minutes. In total, 12 powder and 12 film samples of α-Fe_2_O_3_ were prepared. An additional set of samples prepared by heating γ-FeOOH at 350 °C for 2 hours to obtain Raman spectra for comparison and confirm γ-Fe_2_O_3_ as an intermediate in the transition from γ-FeOOH to α-Fe_2_O_3_.

### Raman spectroscopy

Measurements were carried out on a Renishaw inVia Reflex system in which a microscope is equipped with an encoded sample stage. Measurements were performed using 532 (Renishaw DPSSL laser, 50 mW) and 633 nm (Renishaw HeNe laser, 17 mW) excitation. Data shown in the manuscript was acquired using 633 nm filtered to 5% of maximum intensity in conjunction with an 1800 lines/mm grating, unless otherwise stated. Raman spectra were analyzed using the Renishaw WiRE 5.2 software package. Data processing included baseline subtraction (sample shown in Fig. S1†) and spectrum normalization.

### Infrared spectroscopy

Fourier-transform infrared (FTIR) spectroscopic measurements were performed on a Nicolet 6700 FTIR equipped with a Pike Technologies VeeMAX III variable-angle reflection accessory and a DTGS detector. Spectra were collected with an incident angle of 50°. Cleaned FTO and FTO/ABS were used as the baseline for thin film samples. Spectra are shown in Fig. S2.†

### X-ray diffraction

Powder X-ray diffraction experiments were carried out using Cu Kα radiation on a PANalytical Empyrean diffractometer. Experiments scanned 2*θ* from 10° to 90° at a rate of 5° min^−1^.

### UV-vis spectroscopy

Ultraviolet-visible (UV-vis) absorption of the samples was recorded on a PerkinElmer Lambda 1050 UV-vis spectrophotometer equipped with an integrating sphere. Data was collected in a wavelength range of 200 nm to 1100 nm.

### X-ray photoelectron spectroscopy

X-ray photoelectron spectroscopy (XPS) measurements were performed on a Thermo-VG Scientific ESCALab 250 microprobe using an aluminum X-ray source. Survey scans were performed using a pass energy of 50 eV and high-resolution scans with 30 eV. The X-ray beam was performed at the current of 7.5 mA and the accelerating potential of 15 kV. All measurements were performed on films deposited on FTO glass with a small strip of conductive carbon tape to make electrical contact. Films were thoroughly rinsed with isopropanol and dried with N_2_ gas before loading into the sample chamber. Quantitative analysis of high-resolution scans of Fe 2p_3/2_ and O 1s region was operated by CasaXPS 2.3.19. Spectra were calibrated by shifting the C 1s signal of adventitious hydrocarbons to 285.0 eV binding energy.

### Photoelectrochemical analysis

Experiments were carried out using a Bio-logic SP300 potentiostat and a Sciencetech A1 Lightline solar simulator equipped with an AM1.5G filter. Hematite-coated FTO working electrodes were mounted as windows in a polyethylene cell with the area of the electrode exposed to electrolyte solution masked to 1.54 cm^2^ by a silicone O-ring. A Gaskatel HydroFlex Reversible Hydrogen Electrode (RHE) served as the reference electrode, a piece of clean FTO as the counter electrode, and 1 M KOH as the electrolyte. Photoelectrocatalytic tests utilized back-side illumination and current densities represent the geometric surface area.

## Results

### Characterization of α-Fe_2_O_3_ powders

**Fig. 1:**
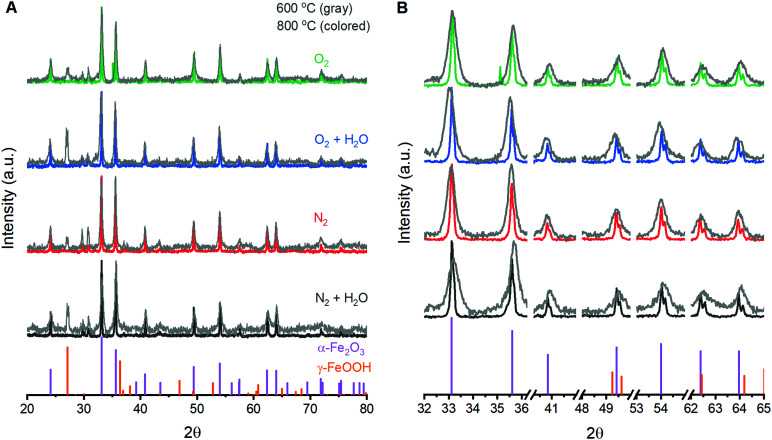
Powder X-ray diffraction patterns obtained following annealing of γ-FeOOH at 600 °C and 800 °C for two hours. The (A) full range and (B) selectively enlarged regions are shown for comparison. The vertical purple and orange lines at the bottom of each panel depict α-Fe_2_O_3_ (COD# 9000139) and γ-FeOOH (COD# 9015231), respectively.

The ability to fabricate α-Fe_2_O_3_ with varied structural integrity by annealing γ-FeOOH under varied atmospheres was explored in the powder form due to ease of fabrication and structural characterization. Powder X-ray diffraction (XRD) experiments confirm that γ-FeOOH is the predominant iron phase obtained through controlled oxidation of aqueous Fe^II^ solutions (Fig. S3†).^29^ The transition of γ-FeOOH to α-Fe_2_O_3_ is a dehydroxylation reaction that proceeds through γ-Fe_2_O_3_ as an intermediate phase, where both hydrogen and oxygen are removed from the structure.^32^ The initial transition occurs above 300 °C;^32^ the powders here yield the characteristic reflections for γ-Fe_2_O_3_ and complete loss of reflections attributable to γ-FeOOH when annealed at 350 °C. Annealing at 600 °C yields the diffraction pattern of α-Fe_2_O_3_ for all four annealing atmospheres, albeit with substantially broadened peaks that indicate significant structural disorder and a peak at *ca.* 27° suggesting residual γ-FeOOH (Fig. 1). Increasing the temperature to 800 °C decreases the XRD peak widths but no differences in the location or width of peaks are detectable between samples annealed at 800 °C under any of the four different atmospheres. Small peaks at 29.7 and 30.7° that persist in the powder samples are due to K_2_SO_4_ trapped during the fabrication process. Raman spectroscopy supports this assignment and confirms its absence in thin film samples (Fig. S4†). The XRD results suggest that, irrespective of the reaction atmosphere, γ-FeOOH undergoes complete conversion to γ-Fe_2_O_3_ at 350 °C, to a disordered form of α-Fe_2_O_3_ at 600 °C, and finally to a more crystalline form of α-Fe_2_O_3_ at 800 °C.

**Fig. 2:**
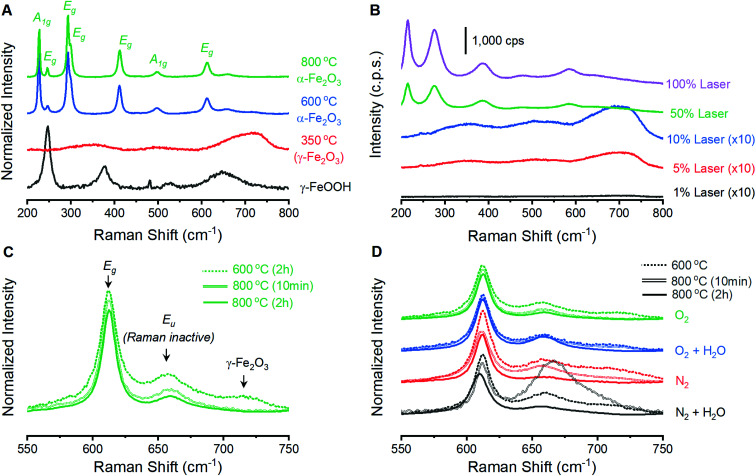
Raman spectra acquired on iron oxide powders. (A) Data acquired on the precursor γ-FeOOH powder and samples annealed at various temperatures for 2 hours under a dry O_2_ atmosphere. (B) Spectra acquired as a function of laser power on a powder annealed at 350 °C under dry O_2_. The intensity of the lower three spectra are increased 10-fold to compensate for the weak scattering cross section of γ-Fe_2_O_3_; a clear change in surface morphology and color is observed following acquisition of the top two spectra. (C) Magnified view of secondary peaks for powders annealed under a dry O_2_ environment. (D) Magnified view of the same region for all 12 powder samples. Panels A, C and D were normalized to the intensity of the 293 cm^−1^ E_g_ vibrational mode.

Raman spectroscopy on powder samples provides evidence that the integrity of the α-Fe_2_O_3_ crystal lattice is in fact affected by atmospheric conditions during annealing. Raman spectra on as-prepared γ-FeOOH powders contain the expected vibrations at 247, 379, 525 and 645 cm^−1^ (Fig. 2A).^33^ These features are replaced by the characteristic peaks for γ-Fe_2_O_3_ at *ca.* 348, 498 and 707 cm^−1^ after annealing at 350 °C for 2 hours, confirming XRD indications of complete structural conversion at these temperatures.^33^ Annealing at 600 or 800 °C results in the emergence of seven intense peaks in the Raman spectrum (Fig. 2A). These well-documented peaks are assigned to the two A_1g_ (226 and 499 cm^−1^) and five E_g_ (246, 293, 412, 497 and 612 cm^−1^) Raman active vibrational modes for α-Fe_2_O_3_.^33–37^ It is notable that these α-Fe_2_O_3_ peaks are orders of magnitude more intense than those for γ-FeOOH and γ-Fe_2_O_3_, and that care must be taken in selecting laser intensity as these phases readily transition into defective α-Fe_2_O_3_ under laser irradiation (Fig. 2B). In addition to the seven expected α-Fe_2_O_3_ vibrational modes, spectra on powders annealed at 600 and 800 °C contain weak peaks at 660 and 710 cm^−1^. The relative intensity of each of these two features is sensitive to annealing protocols (Fig. 2C). Comparisons across the 12 different spectra show that both peaks diminish in size when annealed at higher temperatures or for longer times (Fig. 2D). Accordingly, samples annealed at 800 °C for 2 hours show the smallest 660 cm^−1^ peak in each series and no discernible peak at 710 cm^−1^. All iron oxide crystal structures exhibit vibrations in the 600–700 cm^−1^ region,^33^ making it plausible that the two unexpected features signify phase impurities. The identity of the 660 cm^−1^ peak has been discussed in the literature, with consensus being that this feature is an infrared active (*i.e.* Raman inactive) E_u_ vibrational mode that becomes observable in Raman spectra due to distortions in the crystal lattice.^34,38–40^ An infrared-active vibration is observed in the thin film samples here using FTIR reflection-absorption spectroscopy (Fig. S2†), leading us to follow literature precedent and assign the 660 cm^−1^ to the formally Raman inactive E_u_ vibrational mode and attribute the 710 cm^−1^ feature to γ-Fe_2_O_3_. Raman spectroscopy thus provides a means to track structural distortions in α-Fe_2_O_3_ and confirm that structural integrity is systematically dependent on annealing conditions.

### Raman spectroscopy of photoanode films

**Fig. 3:**
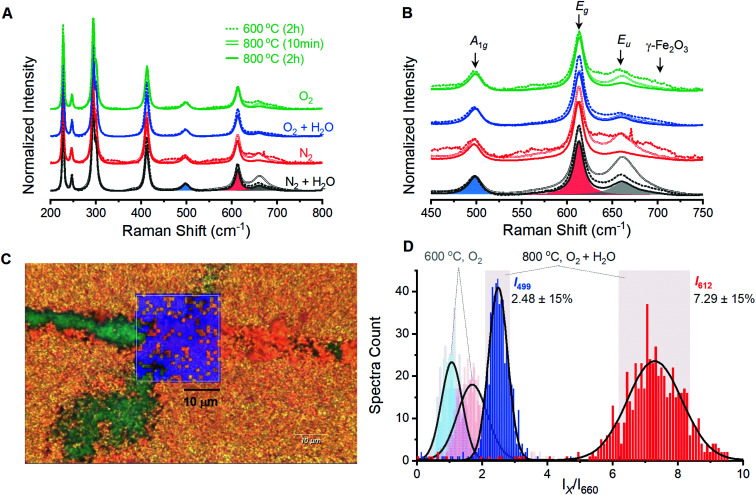
Raman spectra acquired by averaging spectra measured over a 2 × 2 μm grid on α-Fe_2_O_3_ films fabricated under varied atmospheric conditions. The (A) full spectral range and (B) magnification of key peaks, with sample component peaks shaded. (C) A Raman spectroscopic map of the 23 × 24 μm region with blue shading indicating pixels where the *I*_499_/*I*_610_ was within 15% of the histogram maximum. (D) Histograms showing intensity ratios of the A_1g_ (*I*_499_) and E_g_ (*I*_612_) vibrational modes to the E_u_ (*I*_660_) for Raman spectra acquired across a larger 23 × 24 μm region (C) of a film annealed at 800 °C for 2 hours under humidified O_2_ atmosphere. Black curves are a fitted Gaussian distribution and gray shaded areas denote 15% variation from the peak in either direction. Faded histogram at low ratios represents the distributions observed for a sample heated at 600 °C for 2 hours under dry O_2_ atmosphere.

Films prepared by annealing electrodeposited γ-FeOOH under varied environments show behavior analogous to the powder samples. A series of 12 films prepared with annealing conditions identical to the powder samples were analyzed by spectroscopic mapping to ensure that Raman spectra are representative of the entire surface. Spectra were acquired in 1-micron steps across three distinct 2 × 2 micron grids for each of the 12 films; each set of 27 spectra was averaged and normalized (Fig. 3A). These 12 spectra are dominated by the characteristic peaks for α-Fe_2_O_3_, with secondary peaks once again present at *ca.* 660 and 710 cm^−1^ (Fig. 3B). The presence of a 710 cm^−1^ peak in all four films annealed at 600 °C reveals that an incomplete reaction yields γ-Fe_2_O_3_ contamination. The 660 cm^−1^ is again observed to decrease as temperature or annealing time are increased. Only the eight 800 °C samples were selected for detailed analysis as phase contamination in the 600 °C samples would impede clean comparisons between the spectroscopic and electrochemical behavior. The spectra were fitted with 8 component curves (7 Raman active and 1 inactive) to obtain component peak locations, intensities and widths for comparison with electrochemical behavior (Table S1†).

Spectroscopic maps acquired over large areas on select samples provide confidence that samples prepared at 800 °C are homogeneous and yield consistent spectra. All eight samples show the same qualitative behavior; the sample annealed at 800 °C for 2 hours under humidified O_2_ provided the highest photoelectrocatalytic activity (see below) and will be discussed. Visual inspection under magnification shows a polycrystalline orange surface with a small number of green streaks that were identified as bare FTO (Fig. S5†). A region containing an exposed FTO streak was selected for analysis and a series of Raman spectra were collected in 1-micron steps across a 23 × 24 micron grid (Fig. 3C). The reproducibility of spectra acquired by the mapping protocol is confirmed by the superimposition of the average of these 600 spectra with that from the smaller 2 × 2 micron grid (Fig. S6†).

Spectroscopic maps showing the location, intensity and width of each individual peak failed to reveal any irregularities in the α-Fe_2_O_3_ spectra. The ratio of intensities of the 499 and 612 cm^−1^ components to that of the Raman inactive peak at 660 cm^−1^ (*I*_499_/*I*_660_ and *I*_612_/*I*_660_, respectively) were identified to be of importance and are discussed below. Histograms for each of these terms show a Gaussian distribution with *ca.* 80% of the total spectral variation residing within 15% of the distribution maxima (Fig. 3D). We therefore adopt ±15% of the maximum as an error estimate for the structure–property correlations below. Pixels on a surface image that fall outside of this 15% range are randomly scattered on spectroscopic maps (Fig. 3C). This visual confirmation provides further confidence in the uniformity of the surfaces and the viability of using the acquired spectra to represent the overall structure in structure–property analyses.

### Parametrizing photoelectrocatalysis

**Fig. 4:**
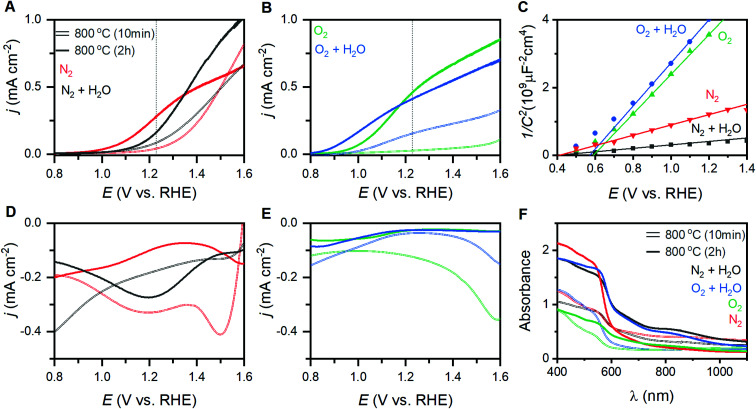
Photoelectrochemical behavior and characterization of α-Fe_2_O_3_ films. Voltammetric behavior under illumination for films annealed under dry and humidified (A) N_2_ and (B) O_2_ environments. (C) Mott–Schottky plots of films prepared by annealing in different conditions at 800 °C for 2 hours. Rapid cathodic sweeps performed in the dark after equilibration at 2 V_RHE_ under illumination for films annealed under dry and humidified (D) N_2_ and (E) O_2_ environments. (F) UV-visible absorption spectra for the eight films annealed at 800 °C.

The eight different samples prepared at 800 °C yield markedly different photoelectrochemical behavior. Voltammetric sweeps while under illumination with artificial sunlight reveal pronounced changes in the onset of photoelectrocatalysis, maximum current densities attained, and even curve shape for each of these α-Fe_2_O_3_ films (Fig. 4A and B). Hematite photoanodes annealed at 350–600 °C are typically found to exhibit low crystallinity and/or incomplete phase transitions.^28^ The samples annealed at 600 °C here suffer from this issue, resulting in unmeasurable PEC performance. The current density at 1.23 V (*j*_1.23V_), or zero overpotential for OER, is a performance metric widely reported in the literature; values for the samples annealed at 800 °C are provided in Table 1.^41,42^ The onset of PEC (*E*_PEC_) was selected as a second performance metric. The voltage at which derivative d*j*/d*E* plots reaches 0.2 mA cm^−2^ V^−1^ has been reported as a means of systematically extracting a numeric value for *E*_PEC_.^43^ This approach works well for samples exhibiting good PEC performance, but fails to capture the onset for poorly performing samples. The first positive peak in second derivative d^2^*j*/d*E*^2^ plots was therefore chosen as a means to systematically represent *E*_PEC_ (Fig. S7†). This approach captures the foot of the catalytic wave, thereby generalizing the metric such that it is not coupled to specific performance values.

**Table 1:** Behavioral parameters for α-Fe_2_O_3_ films prepared at 800 °C

Condition	*j*_1.23V_ (mA cm^−2^)	*E*_PEC_ (V *vs.* RHE)	*E*_fb_ (V *vs.* RHE)	*E*_g_ (eV)
N_2_ + H_2_O, 10 min	0.08	1.293	0.601	2.06
N_2_, 10 min	0.04	1.390	0.310	2.07
O_2_ + H_2_O, 10 min	0.18	1.012	0.420	2.06
O_2_, 10 min	0.02	1.004	0.390	2.07
N_2_ + H_2_O, 2 h	0.16	1.215	0.392	2.05
N_2_, 2 h	0.36	1.095	0.410	2.08
O_2_ + H_2_O, 2 h	0.41	0.878	0.575	2.06
O_2_, 2 h	0.48	1.029	0.591	2.05

### Band structure measurements

The composition of the annealing atmosphere systematically affects the electronic band structure. Electrochemical impedance spectroscopy (EIS) performed between 0.5 to 1.5 V_RHE_ on each hematite film provides both capacitance and resistance values (Fig. S8†). Mott–Schottky analysis using the capacitance values (Fig. 4C) indicates that the flat band potentials (*E*_fb_) for the α-Fe_2_O_3_ conduction band vary between 0.31 and 0.60 V_RHE_ (Table 1). Free charge carrier concentrations (*N*_d_) calculated from the slope of the Mott–Schottky plots (Table S2†). Peaks observed during rapid cathodic sweeps (performed in the dark) after holding photoelectrodes at catalytic potentials while under illumination have been shown to reveal the location of intraband states.^26,44^ Transient absorbance spectroscopy, EIS, and X-ray absorption studies provide support for such an assignment.^3,26,45–48^ Rapid sweep experiments on the thin films here yield cathodic peaks at voltages that correspond to photoelectrocatalytic onset (Fig. 4D, E and S9†). The weak intensity and width of these features make it difficult to systematically extract meaningful numeric values for comparison. Following the aforementioned relationship between these states and the onset of photoelectrocatalysis, we use *E*_PEC_ as a proxy for the location of these intraband states as it can be clearly defined and systematically extracted. We note that while examining *E*_PEC_ behavior as a function of other material parameters grants insight into the intraband states, the term should not be viewed as a quantitatively accurate measure for the location of these states. UV-visible spectra yield optical band gaps between 2.05 and 2.08 eV (Fig. 4F, Table 1), with an average of 2.06 ± 0.02 eV across all eight samples.

### Correlating structure and behavior

**Fig. 5:**
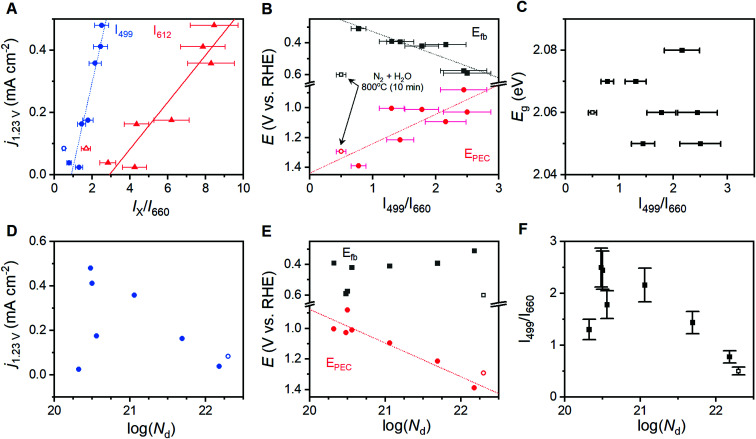
Correlations observed between Raman spectra and properties across the sample series. Correlations are shown for the relative intensity of key Raman vibrations and (A) photocurrent density at 1.23 V *vs.* RHE, (B) the onset of photoelectrocatalysis and the flat-band potential, and (C) optical band gap. Correlations between charge carrier concentration, *N*_d_, and (D) photocurrent density at 1.23 V *vs.* RHE, (E) flat band potential and onset of photoelectrocatalysis, and (F) Raman intensity ratio. Error bars shown depict ±15% of the average Raman peak intensity ratio. Trend lines did not consider the sample annealed under humidified N_2_ atmosphere for 10 minutes (hollow data points) as it exhibits a different electronic structure (see text).

Comparisons of the comprehensive Raman spectroscopy dataset with the extracted metrics show that the *I*_499_/*I*_660_ and *I*_612_/*I*_660_ ratios have special significance. A positive correlation exists between the catalytic metric *j*_1.23V_ and both *I*_499_/*I*_660_ and *I*_612_/*I*_660_ (Fig. 5A). Similar correlations are not observed for other peak intensities (Table S1†). The lattice distortion that makes the E_u_ vibrational mode at 660 cm^−1^ visible in a Raman spectrum must therefore uniquely affect the high energy A_1g_ and E_g_ vibrations; the magnitude of these distortions, in turn, inhibits photoelectrocatalytic performance. Comparison of *I*_499_/*I*_660_ and *I*_612_/*I*_660_ with other component peak parameters reveals exponential relationships with the full-width at half mass of 5 Raman active vibrations (Fig. S10†), but no clear correlations with peak positions. The *I*_499_/*I*_660_ ratio correlates positively to measured *E*_fb_ values for the α-Fe_2_O_3_ conduction band, with an anodic shift of *ca.* 145 mV per ratio unit, but negatively to the *E*_PEC_ values that serve as a proxy for the location of intraband states (Fig. 5B). The optical band-gap is essentially unchanged across the sample series (Fig. 5C). No clear correlation is observed between *N*_d_ values and either *j*_1.23V_, *E*_fb_ or *I*_499_/*I*_660_ (Fig. 5D–F). The *E*_PEC_ values, however, shift anodically with increased *N*_d_.

**Fig. 6:**
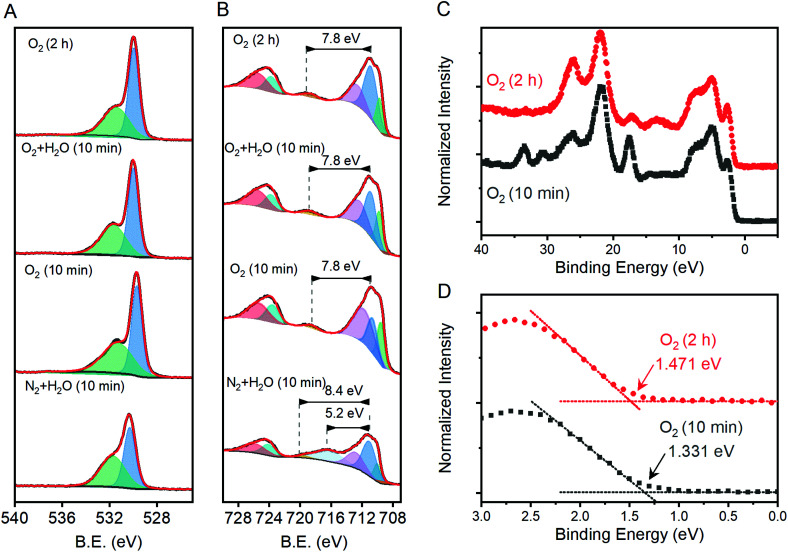
High resolution X-ray photoelectron spectroscopy results in the (A) O 1s and (B) Fe 2p and (C) valence band regions for selected samples annealed at 800 °C. (D) A magnification of the valence band edge region highlighting differences between samples annealed in O_2_ for 2 h and 10 min. Panels A and B show thick black lines for raw data, component peaks used to fit the data as shaded peaks and the sum of all component peaks as thin red lines.

X-ray photoelectron spectroscopy was performed on a subset of samples, with three selected to span the range of measured *j*_1.23V_ values and one being the apparent outlier. The O 1s region shows components at *ca.* 531.7 and 530.0 eV that are attributable to OH^−^ and O^2−^ species, respectively (Fig. 6A). The introduction of oxygen vacancies into α-Fe_2_O_3_ has been shown to increase the relative intensity of the high energy component by a factor of 3.^21^ The relative intensity of the high energy O 1s component here does not exhibit significant changes for the three samples that lie on structure–property trend lines in Fig. 5; the relative intensity of the feature does decrease for the sample that was annealed for 10 min at 800 °C under humidified N_2_, which is an outlier in structure–property trends. The existence of a low energy shoulder on the Fe 2p_3/2_ peak has been previously used to support the presence of oxygen vacancies by observation of associated Fe(ii) within α-Fe_2_O_3_,^21^ but detailed XPS studies show that electronic structure of Fe(iii) based materials typically yield such features.^49^ The energy spacing between the primary Fe 2p_3/2_ peak and the shake-up satellite peak provides a more reliable means to differentiate between Fe(iii) and Fe(ii), with spacing for the former being *ca.* 8 eV and the latter *ca.* 5.5 eV for iron oxides.^49^ A consistent 7.8 eV spacing is observed here for the three samples that fall on the structure–property trend lines while the outlier exhibits a strong satellite peak located 5.2 eV above the Fe 2p_3/2_ peak (Fig. 6B). High resolution spectra show that the valence band edge location shifts from *ca.* 1.331 eV for the sampled heated in O_2_ for 10 min to 1.471 eV for that heated in O_2_ for 2 hours (Fig. 6C and D; C 1s calibrated to 285.0 eV). Comparison of these values with respective *I*_499_/*I*_660_ ratios suggests that the valence band shifts anodically at a rate of *ca.* 116 meV per ratio unit. These results suggest that the annealing environment yields negligible changes in the average Fe oxidation state for samples that lie on the trend line, but a measurable amount of Fe(ii) ions in the sample that is an outlier in all structure–property trends.

## Discussion

### Control over structural integrity

A series of 12 α-Fe_2_O_3_ powders prepared by annealing γ-FeOOH under varied conditions confirm that the integrity of the crystal lattice is affected by the temperature, heating time and annealing atmosphere. Powder X-ray diffraction experiments reveal α-Fe_2_O_3_ in all samples, but samples annealed at 600 °C exhibit broad XRD peaks (Fig. 1). All samples prepared at 800 °C yield well-defined diffraction patterns with no clear variations arising due to changes in annealing time or atmospheric composition. Raman spectra from all 12 samples contain the 7 Raman active vibrational modes expected for α-Fe_2_O_3_ plus two unexpected peaks: a peak at 710 cm^−1^ attributed to an incomplete decomposition product (γ-Fe_2_O_3_) and a peak at 660 cm^−1^. This latter feature has been previously assigned to an infrared active E_u_ vibrational mode for α-Fe_2_O_3_ that becomes observable in Raman spectra when structural distortions break lattice symmetry.^34,36,50,51^ FTIR measurements here support such an assignment (Fig. S2†). The exact nature of the distortion, however, remains unclear. Annealing conditions that are expected to improve the quality of the α-Fe_2_O_3_ lattice – increased temperatures, longer reaction times and the absence of atmospheric H_2_O – all decrease the intensity of the 660 cm^−1^ peak (Fig. 2). The intensity of this distortion-induced vibration thus provides a numeric indicator of structural quality by describing the extent of an as-yet unidentified lattice distortion in α-Fe_2_O_3_. This peak grants the ability to extract insights into the nature of the lattice distortion and its effect on PEC performance through structure–property analyses.

An analogous series of twelve α-Fe_2_O_3_ films prepared from electrodeposited γ-FeOOH served as the samples for structure–property analysis. Spectra acquired on the film samples mirror those from powder samples, with the exception of the 710 cm^−1^ peak being unobservable for film samples annealed at 800 °C. The otherwise similar trends and intensities between the powder and film samples suggest that inadvertent doping of α-Fe_2_O_3_, for example *via* leeching from the transparent conducting oxide substrates,^27^ is not occurring here. Further evidence to support this claim comes from previous reports indicating concerted increases in *j*_1.23V_ and the intensity of the 660 cm^−1^ Raman vibration as the concentration of silicon dopant in α-Fe_2_O_3_ films is increased,^38^ which is opposite to the trends observed here. The *I*_499_/*I*_660_ and *I*_612_/*I*_660_ peak intensity ratios from spectroscopic maps of the film samples can confidently be taken as numeric indicators of structural quality for the film samples.

### Influence on PEC

A linear correlation between Raman intensity ratios and *j*_1.23V_ signifies that progressive distortion of the hematite lattice directly inhibits photoelectrocatalytic OER in the sample series (Fig. 5A). The remaining correlations in Fig. 5 provide insight into the fundamental causes for this relationship. The measured *E*_fb_ for the conduction band shifts anodically from 0.31 to 0.59 V_RHE_ as *I*_499_/*I*_660_ increases (Fig. 5B), which spans the range of values commonly reported in the literature.^7,16,17^ This shift implies that the observed lattice distortion induces a shift cathodic shift in the Fermi level. As α-Fe_2_O_3_ is an intrinsic n-type semiconductor, the direction and systematic nature of this shift could be the result of (i) an increased concentration of donor sites (*i.e.* an increase in film doping), (ii) a broadened energy distribution of donor states, or (iii) a shift in the energy level of the donor sites and/or semiconductor band edges.

A filling or broadening of donor states may be expected to shift *E*_fb_ cathodically while simultaneously increasing *N*_d_ values. Increased *N*_d_ values resulting from the introduction of oxygen vacancies have been reported to increase current densities for α-Fe_2_O_3_ photoanodes by providing catalytically active sites for OER, but shift *E*_PEC_ values anodically due to increased recombination rates.^23^ A correlation is observed here between *N*_d_ values and *E*_PEC_, but a failure to observe a clear relationships between *N*_d_ and *j*_1.23V_ or *E*_fb_ (Fig. 5E) suggests that a simple broadening or filling of donor sites is unlikely. A temperature-induced decrease in crystallinity has been demonstrated for zinc ferrite photoanodes, where higher temperatures increased structural disorder that induced a 10-fold increase in bulk resistance and increased the rate of bulk recombination.^52^ Electron transfer resistance values obtained from EIS results on the α-Fe_2_O_3_ films studied here show some variation in resistance across the sample series, and again show no discernible relationship to Raman intensity ratios (Fig. S8†) or *j*_1.23V_ values. A convolution of multiple effects cannot be decisively ruled out, but the linear correlation between *I*_499_/*I*_660_ and *j*_1.23V_ suggests that there is a single factor that dominates PEC behavior across this sample series. The lack of definitive trends between *N*_d_ and important material properties suggests that a broadening or filling of donor states is unlikely to be responsible for the behavior observed here.

Shifting of band edge locations and/or the location if intraband states would change the thermodynamics for electron transfer reactions and alter the kinetics of all processes, including photoelectrochemical OER and recombination. A constant optical band gap of *ca.* 2.06 eV across the sample series necessitates that any shift in the conduction band location be mirrored by a shift in the valence band location. Measured *E*_fb_ values shift at a rate of *ca.* 145 mV per unit of *I*_499_/*I*_660_ (Fig. 5B); XPS results suggest that an increased lattice distortion magnitude causes the valence band to shift anodically at a comparable 116 mV per unit. The rapid voltammetric sweep protocol employed here previously revealed peaks between 0.7 and 1.3 V_RHE_ in α-Fe_2_O_3_ photoanodes that were assigned to defect states that were either (i) catalytic intermediates for OER or (ii) catalytically inactive defects that pin the Fermi level until depletion by a sufficient voltage.^26,45–48^ Taking *E*_PEC_ as a proxy for the location of the intraband states, as has been previously argued,^26,45–48^ the defect states can be viewed as shifting cathodically from *ca.* 1.38 V_RHE_ in the most distorted and worst performing samples to 0.88 V_RHE_ in the best performing samples (Fig. 5B). The location of these peaks allows us to rule out the possibility that the species observed here are reaction intermediates, or otherwise directly responsible for catalyzing OER, on thermodynamic grounds. We propose instead that the observed *E*_PEC_ values approximate the location of intraband defects that act as recombination sites. The correlations of *I*_499_/*I*_660_ with both *E*_fb_ and *E*_PEC_ thus indicate a concurrent anodic shift in the defect states and cathodic shift in the semiconductor band edges. These opposing shifts would have two effects on catalysis: (i) relaxation of photoexcited electrons would be facilitated by the movement of recombination sites towards the midpoint in energy of the two bands, and (ii) the cathodic shift in the valence band location would decrease the thermodynamic driving force to use photoexcited holes in the valence band to drive OER.

### Nature of the defect

Oxygen vacancies within the lattice are unlikely to be responsible for the behavior observed here. Annealing under N_2_ environments has been widely reported as a means to generate oxygen vacancies in α-Fe_2_O_3_ photoanodes, which increases the charge carrier density and improves photocatalytic performance.^20–24^ Here, we observe consistently improved performance for samples annealed under oxygen environments and no clear correlations between photocurrents and measured charge carrier densities (Fig. 5D; Table S2†). XPS measurements yield evidence for oxygen vacancies and the associated Fe(ii) sites only for the sample that does not match all observed structure–property trends (Fig. 5). The inversion of expected behavior here compared to the trends consistently reported in the literature, and XPS results, lead us to rule out oxygen vacancies as the structural defect of interest here.

**Fig. 7:**
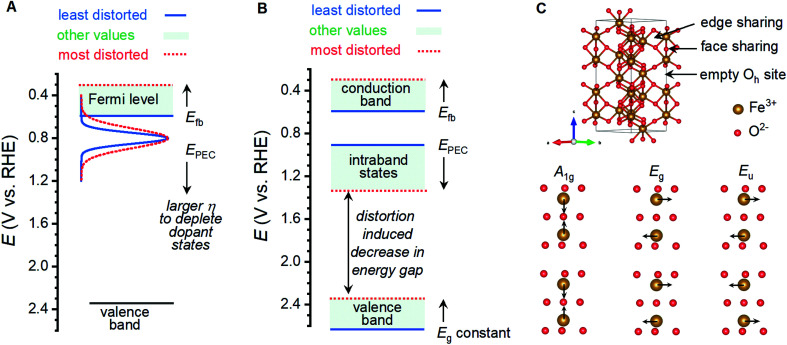
Possible changes in band structure induced by lattice distortion and the identity of critical vibrations for α-Fe_2_O_3_. (A) An increase in concentration of donor states and broadening of donor states may induce a cathodic shift in the Fermi level and necessitate a larger overpotential to initiate photoelectrocatalytic OER. (B) A cathodic shift in conduction and valence band edges and an anodic shift in intraband states may increase the rate of recombination, shifting photoelectrocatalytic OER to voltages where the recombination states are depleted. (C) Unit cell for α-Fe_2_O_3_ denoting the connectivity and key structural features, and the identity of Raman vibrations at *ca.* 499, 612 and 660 cm^−1^ that are found to correlate with photoanode properties.

Analysis of the nature of Raman vibrations leads us to propose that the performance altering distortion observed here is induced by the trapping of protons in the α-Fe_2_O_3_ lattice. Hematite adopts the corundum crystal structure, where Fe^III^ ions reside in two-thirds of the octahedral sites in a lattice of hexagonally close-packed oxide ions. The crystalline *c*-axis is characterized by columns of alternating dimers of face-sharing Fe^III^ octahedra and vacant octahedral sites, with edge-sharing motifs linking columns together (Fig. 7B). The thermal transformation of goethite (α-FeOOH) to α-Fe_2_O_3_ is known to progress through an intermediate phase termed “protohematite”,^53,54^ where incomplete dehydroxylation results in residual hydrogen content within the solid-state structure. Synchrotron-based XRD on protohematite revealed it to be an Fe-deficient form of α-Fe_2_O_3_ where Fe^III^ vacancies are charge-balanced by protonation of oxygen ions (*i.e.* substitution of oxide with hydroxide; α-Fe_2−*x*/3_(OH)_*x*_O_3−*x*_).^53,54^ Protons within the lattice alter the two unique Fe–O distances in the structure, with those involved the face sharing motifs expanding from 2.11 to 2.15 Å as the concentration of protons increases, and those adjacent to octahedral vacancies contracting from 1.95 to 1.91 Å.^53^ In terms of Fe–Fe distances, protonation (1) contracts face-sharing motifs by 0.067 Å or 2.7%, (2) contracts edge-sharing motifs by 0.025 Å or 0.8%, and (3) expands the distance across octahedral vacancies by 0.037 Å or 0.6%. The A_1g_, E_g_ and E_u_ vibrational modes whose intensities correlate to the physical properties and PEC performance measured here involve the simultaneous movement of Fe^III^ ions linked by the face-sharing motifs (Fig. 7C), while the remaining 5 Raman vibrations that do not correlate with the measured properties involve the simultaneous movements of Fe^III^ linked through edge-sharing motifs, or the movement of oxide ions (Fig. S10†). The observed distortion must therefore involve pronounced changes in the face-sharing motifs, but be largely insensitive to edge sharing motifs. This is consistent with the structural changes between α-Fe_2_O_3_ and α-Fe_2−*x*/3_(OH)_*x*_O_3−*x*_. The exponential relationship between our metric for structural distortion, *I*_499_/*I*_660_, and all vibrations involving movement of oxygen ions can be attributed to protonation of oxide ions, reinforcing this comparison. Confidence in the assignment is further bolstered by the need to decrease the O : Fe ratio on transitioning from γ-FeOOH to γ-Fe_2_O_3_, the observation that humidified atmospheres produce higher *I*_499_/*I*_660_ ratios, and previous reports of a Raman vibration at 665 cm^−1^ in protohematite.^55^ We therefore conclude that the distortion of interest in the present sample series involves the trapping of protons within the crystal lattice, which induces iron deficiency and displaces face-sharing Fe^III^ ions away from each other.

The highly variable performance of α-Fe_2_O_3_ photoanodes for PEC water oxidation is mirrored by significant dispersion in experimentally measured properties.^56,57^ Structural defects are often cited to explain the experimental variability as they are known to exert significant influence over band structure and photophysics. A lack of information regarding the specific chemical nature of these defects inhibits attempts to remove or avoid them. Here, we establish the trapping of protons within the crystal lattice as a structural defect of interest. This defect is challenging to detect by laboratory XRD but is known to induce iron vacancies within the lattice and to distort the bonding framework of α-Fe_2_O_3_.^53,55^ We find that the presence of H_2_O or the exclusion of O_2_ from the atmosphere during α-Fe_2_O_3_ film preparation has a significant impact on the extent of protonation in α-Fe_2_O_3_. The measured flat band potentials, PEC onset potentials, and optical band gaps for the sample series analyzed here span the range commonly found in the literature; this distortion may therefore be a factor that contributes to the reported variability. We note that the trend reported here is opposite to that observed by Grätzel and co-workers for Si-doped α-Fe_2_O_3_ photoanodes fabricated by atmospheric pressure chemical vapor deposition, where increased intensity of a 660 cm^−1^ Raman vibration correlated to increased silicon dopant concentrations and increased photocurrent densities.^38^ The inversion of trends suggests that multiple defects may induce appearance of the E_u_ vibration in Raman spectra, necessitating further efforts to deconvolute the effects of relevant structural defects. Our finding that the *I*_499_/*I*_660_ ratio tracks changes in properties for α-Fe_2_O_3_ nonetheless identifies Raman spectroscopy as a convenient means to both diagnose a specific structural defect and parametrize it for structure–property analyses. We anticipate that this will greatly facilitate efforts to optimize fabrication protocols for defect-free α-Fe_2_O_3_ photoanodes.

## Conclusions

We fabricated a series of α-Fe_2_O_3_ films by annealing γ-FeOOH with varied protocols and analyzed the series through structure–property analysis. Raman spectroscopy was found to be particularly useful in acquiring information regarding bonding within the crystals and a spectroscopic mapping technique was established to obtain quality, reproducible spectra for each sample. A Raman inactive E_u_ vibrational mode observed at 660 cm^−1^ in all samples confirmed a varying degree of crystal lattice distortion across the sample series. The ratios of the A_1g_ (499 cm^−1^) and E_g_ (612 cm^−1^) vibrational modes to the E_u_ vibration were found to correlate to a number of important properties across the sample series, including the photocurrent density at 1.23 V_RHE_, the location of intraband trap states, band gap and flat band potential. Analysis of the nature of these three vibrational modes and the annealing conditions that lead to the smallest distortion and best PEC performance leads us to propose that the PEC-inhibiting distortion is the generation of Fe^III^ vacancies induced by the trapping of protons within the crystal lattice. These defects inhibit PEC reactions by facilitating charge recombination. Our finding of these correlations and trends provides a rapid, simple way in which the community can employ Raman spectroscopy to rationally guide catalyst development or pre-screen the structural integrity of α-Fe_2_O_3_ photoelectrocatalysts.

## Conflicts of interest

There are no conflicts to declare.

## Supplementary Material

SC-011-C9SC04853G-s001
