# Supplementary material for: Identifying protons trapped in hematite photoanodes through structure–property analysis
Source: Chem Sci. 2019 Dec 16;11(4):1085–96. doi: 10.1039/c9sc04853g (PMC8145353; doi:10.1039/c9sc04853g)
Supplement: SC-011-C9SC04853G-s001 [file SC-011-C9SC04853G-s001.pdf]

Electronic Supplementary Material (ESI) for Chemical Science.
This journal is © The Royal Society of Chemistry 2019

Identifying Protons Trapped in Hematite Photoanodes Through Structure-Property Analysis

Yutong Liu, Rodney D. L. Smith[a,b,*]

[a]*Department of Chemistry,University of Waterloo, 200 University Avenue W., Waterloo, Ontario, Canada N2L 3G1*

[b]*Waterloo Institute for Nanotechnology,University of Waterloo, 200 University Avenue W., Waterloo, Ontario, Canada N2L 3G1*

Correspondence to:

rodsmith@uwaterloo.ca

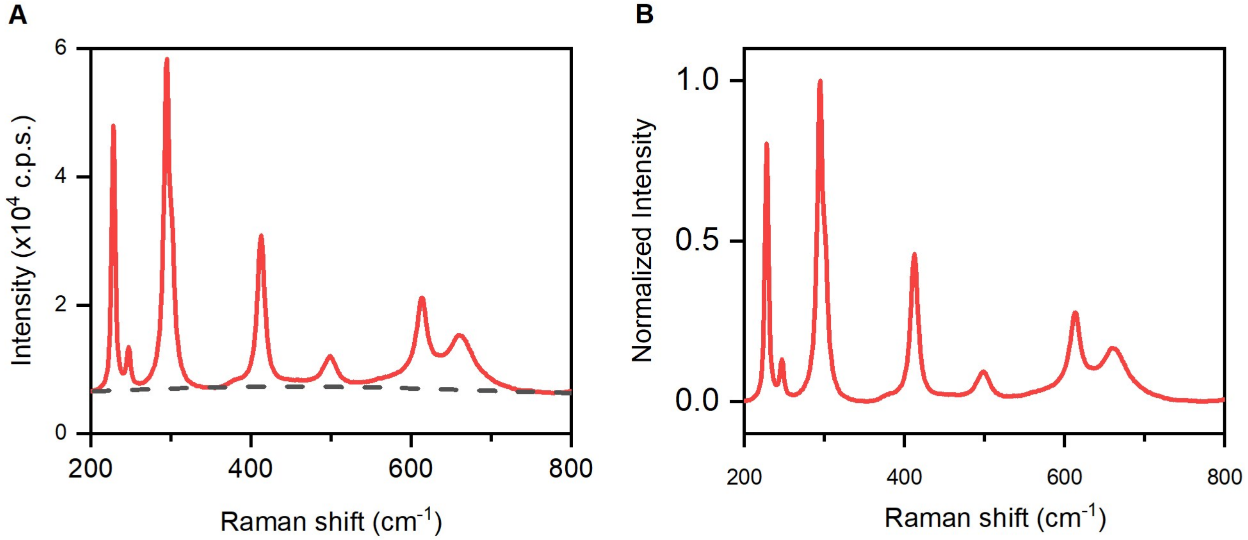

Figure S1. Sample of processing protocol used for Raman spectra showing the sampled annealed at 800 ºC for 10 min. under humidified N_2 environment. (A) The raw spectrum and baseline to be subtracted. (B) Normalized form of the baseline subtracted data.

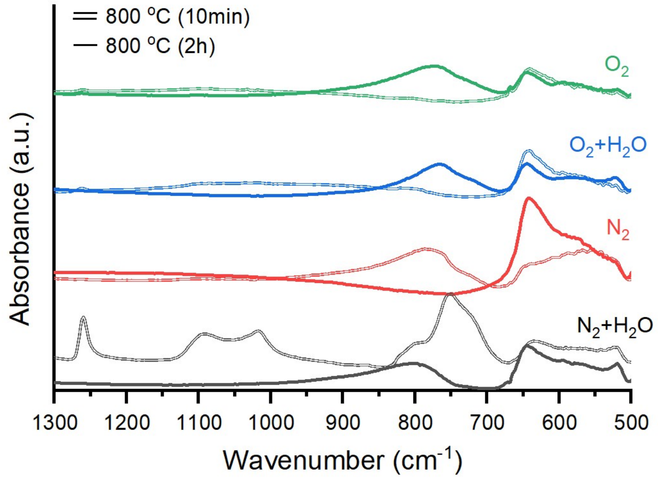

Figure S2. Infrared spectra for hematite thin film samples annealed at 800 °C 10 min and 2h. The sample annealed under humified N_2 for 10 min is an outlier in all structure-property trends.

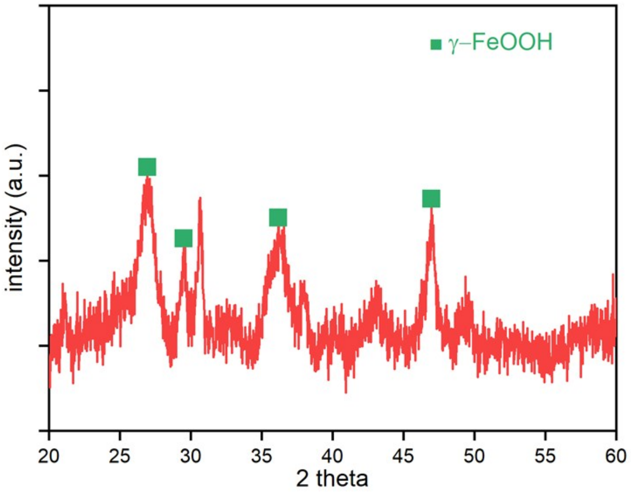

Figure S3. X-ray diffraction pattern obtained on the γ-FeOOH precursor powders.

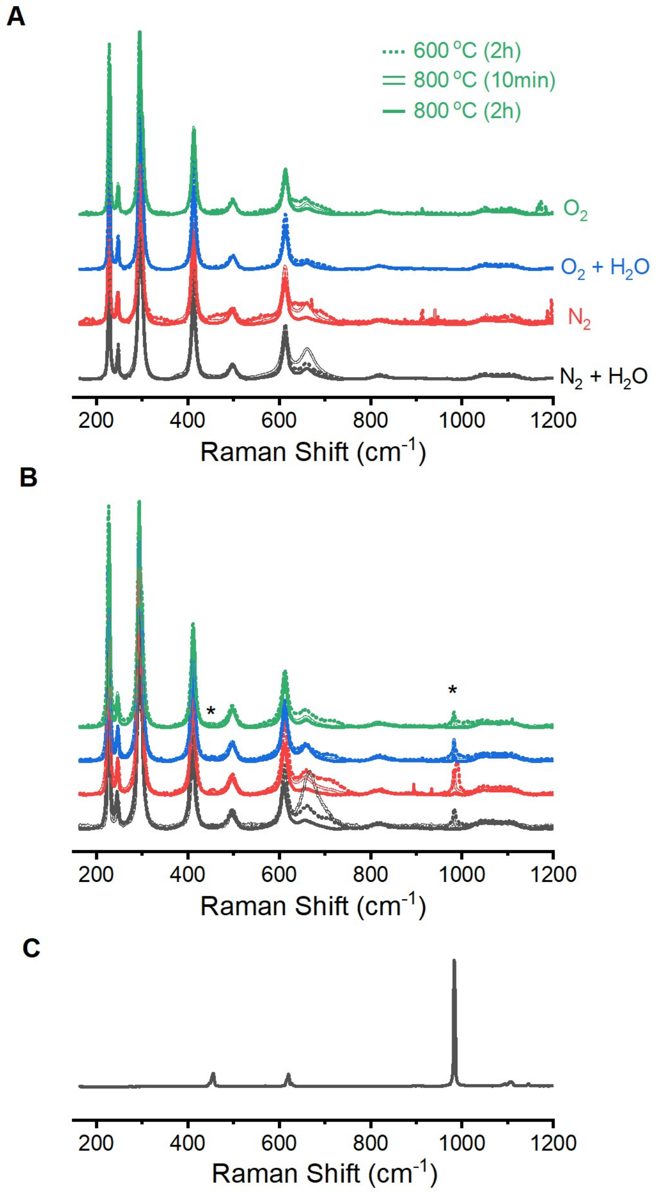

Figure S4. Comparison of Raman spectra for (A) thin film samples and (B) powder samples with (C) K_2SO_4.

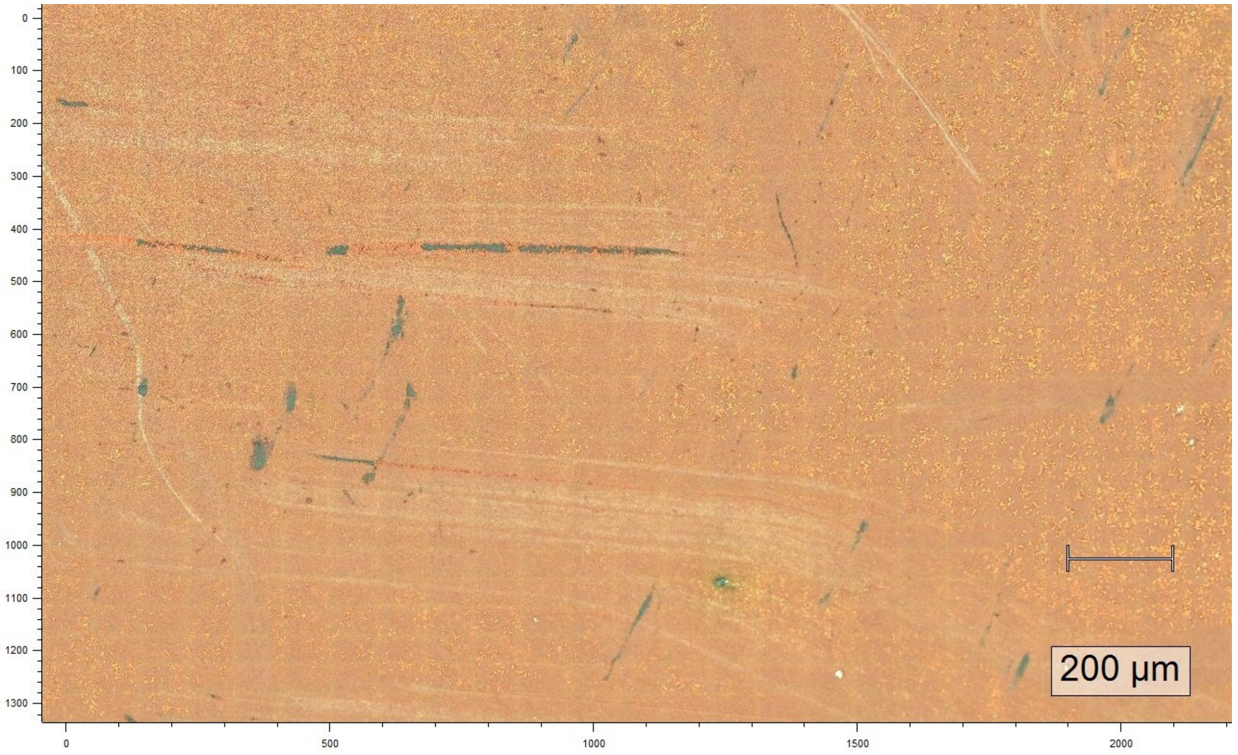

Figure S5. Microscope image of the sample prepared by annealing electrodeposited γ-FeOOH at 800 ^{o}C for hours under humidified O_2 atmosphere.

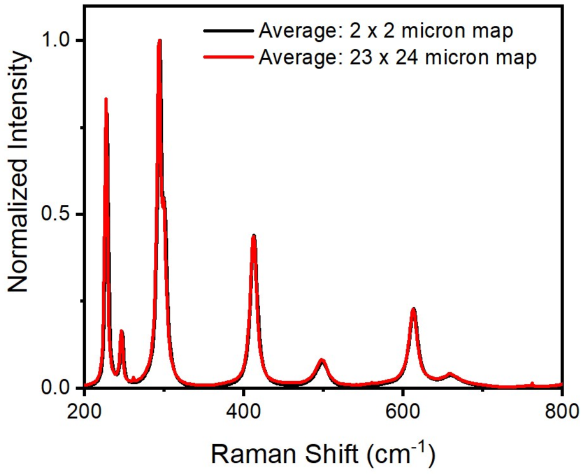

Figure S6. Comparison of the average of all spectra acquired from 2x2 micron squares (27 spectra) and 23 x 24 micron squares (600 spectra).

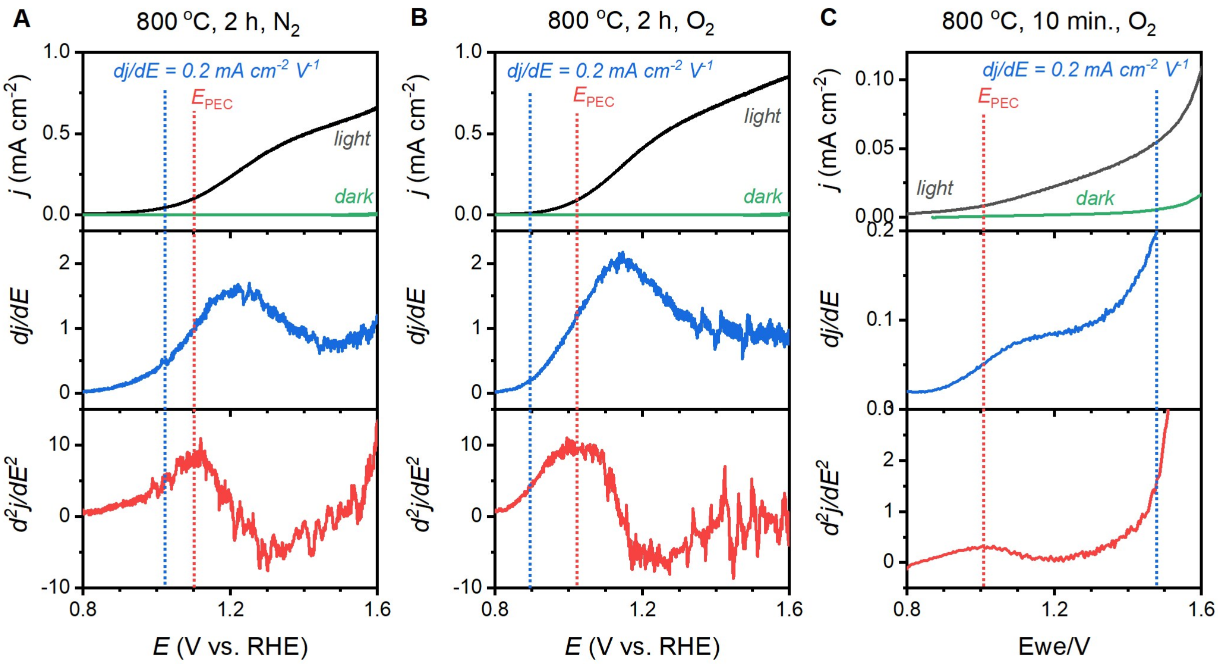

Figure S7. Method for determining photoelectrocatalytic onset. Samples shown are (A) 800 ºC for 2 hours under dry N_2 and (B) 800 ºC for 2 hours under dry O_2, and (C) 800 ºC for 10 min under dry O_2.

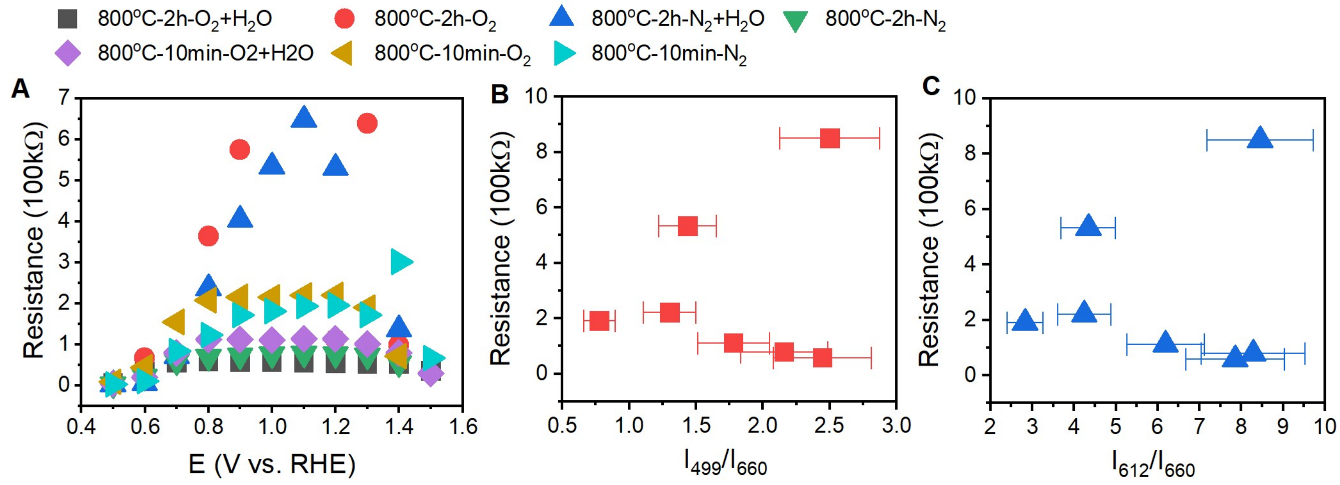

Figure S8. Bulk resistance obtained from fitting EIS data on the α-Fe_2O_3 sample series. (A) Resistance as a function of voltage. Resistance at 1.0 V_{RHE} as a function of Raman intensity ratios (B) I_{499}/I_{660} and (C) I_{612}/I_{660}.

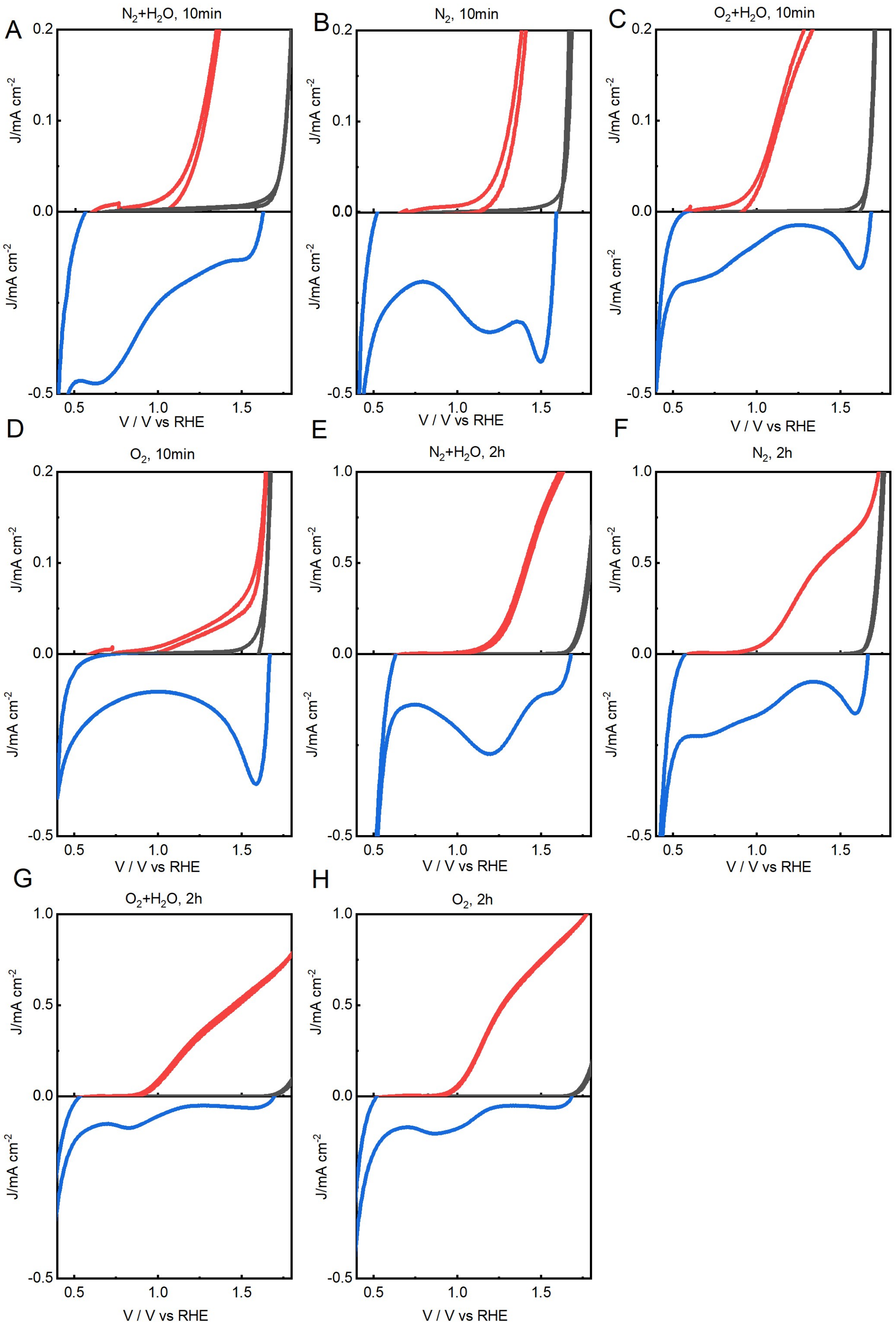

Figure S9. Voltammetric behavior of α-Fe_2O_3 photoanodes in the dark (black lines) and under illumination (red lines), and 1 V s^{-1} cathodic sweeps following equilibration at an oxidizing voltage while under illumination. Data is shown for samples heated at 800 ºC for 10 minutes in (A) humidified N_2, (B) dry N_2, (C) humidified O_2, (D) dry O_2, and at 800 ºC for 2 hours in (E) humidified N_2, (F) dry N_2, (G) humidified O_2, (H) dry O_2.

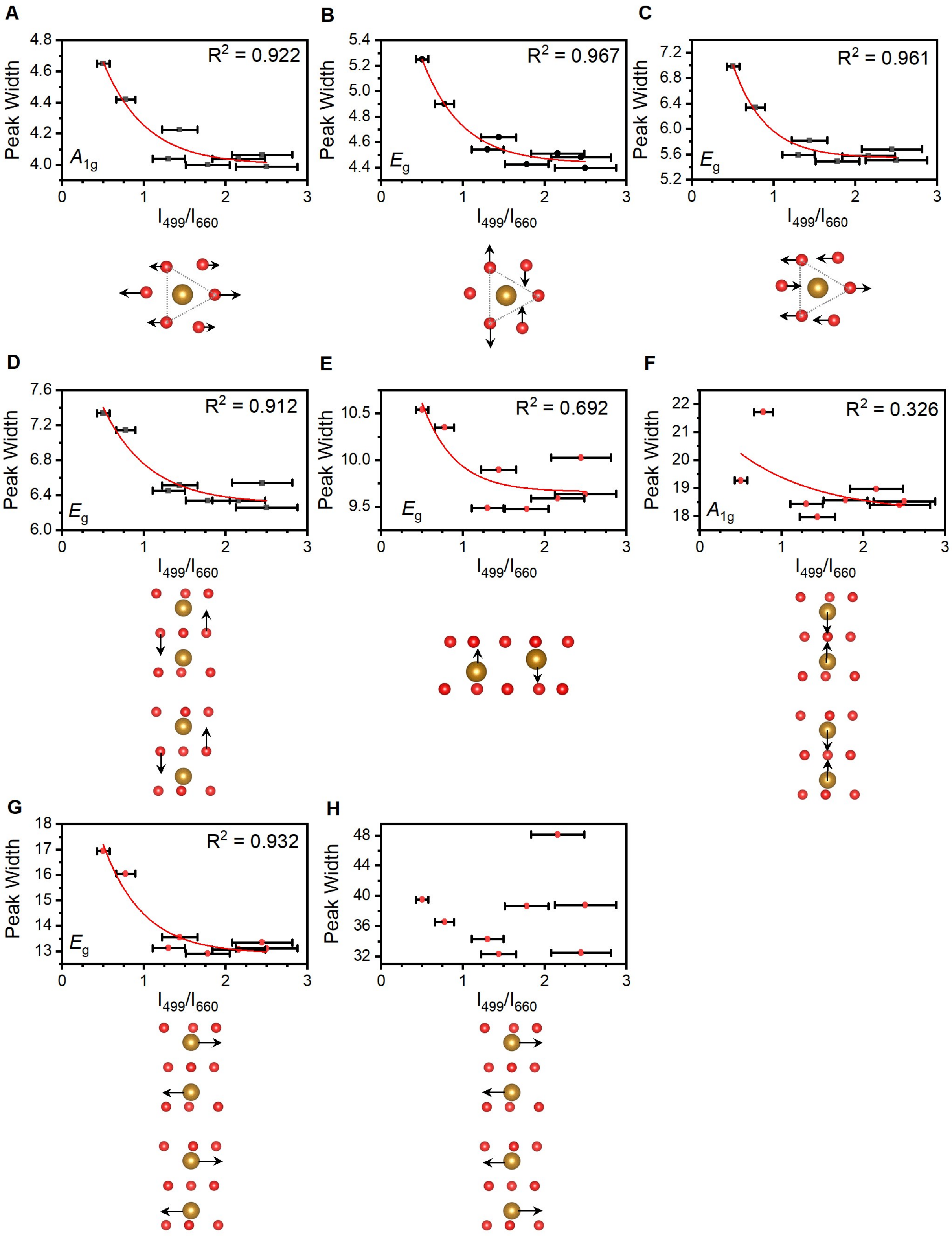

Figure S10. Correlations between peak width and intensity ratio for observed features in the Raman spectra.

Table S1. Location, intensity and width of peak components for the 800 °C α-Fe_2O_3 films.

Sample		A_{1g}	E_g	E_g	E_g	E_g	A_{1g}	E_g	E_u
N_2+H_2O	Centre	228.02	247.04	294.19	301.02	412.40	497.64	612.93	661.95
10min	Width	4.65	5.25	6.98	7.34	10.54	19.28	16.94	39.52
	Height	0.80	0.14	0.93	0.34	0.50	0.08	0.26	0.15
N_2	Centre	227.38	246.65	293.60	300.33	412.26	497.24	612.98	660.36
10min	Width	4.42	4.90	6.34	7.14	10.35	21.72	16.04	36.55
	Height	0.81	0.17	0.91	0.40	0.57	0.08	0.29	0.10
O_2+H_2O	Centre	228.43	247.60	294.56	301.28	413.25	498.10	613.69	662.70
10min	Width	4.00	4.43	5.49	6.34	9.48	18.57	12.91	38.66
	Height	0.85	0.16	0.93	0.39	0.47	0.07	0.23	0.04
O_2	Centre	228.06	247.23	294.27	301.07	413.09	497.79	613.71	661.69
10min	Width	4.04	4.54	5.59	6.45	9.48	18.44	13.12	34.31
	Height	0.83	0.16	0.95	0.40	0.48	0.07	0.24	0.06
N_2+H_2O	Centre	227.96	247.06	294.04	300.86	412.65	498.11	613.05	661.73
2h	Width	4.23	4.64	5.82	6.51	9.90	17.97	13.55	32.29
	Height	0.88	0.14	0.96	0.38	0.44	0.08	0.23	0.05
N_2	Centre	227.51	246.75	293.67	300.35	412.28	496.59	612.62	663.06
2h	Width	4.04	4.51	5.57	6.34	9.59	18.98	13.07	48.11
	Height	0.81	0.17	0.92	0.40	0.51	0.06	0.25	0.03
O_2+H_2O	Centre	228.26	247.39	294.32	301.09	412.93	498.15	613.29	660.97
2h	Width	4.06	4.48	5.68	6.54	10.03	18.40	13.34	32.48
	Height	0.85	0.16	0.95	0.38	0.44	0.07	0.23	0.03
O_2	Centre	228.44	247.60	294.54	301.31	413.18	498.22	613.52	662.74
2h	Width	3.99	4.40	5.51	6.26	9.64	18.53	13.11	38.80
	Height	0.85	0.15	0.94	0.39	0.46	0.07	0.23	0.03

Table S2. Carrier concentration values compared to photocurrent densities for α-Fe_2O_3 films prepared at 600 and 800 °C.

Sample	log(N_d /cm^{-3})	$j_{1.23V}$(mA·cm^{-2})
N_2+H_2O, 600 °C	20.53	0.0078
N_2,600 °C	21.05	0.0045
O_2+H_2O, 600 °C	20.41	0.010
O_2, 600 °C	20.34	0.0026
N_2+H_2O, 10min, 800 °C	22.30	0.08
N_2, 10min, 800 °C	22.18	0.04
O_2+H_2O, 10min, 800 °C	20.56	0.18
O_2, 10min, 800 °C	20.32	0.02
N_2+H_2O, 2h, 800 °C	21.69	0.16
N_2, 2h, 800 °C	21.06	0.36
O_2+H_2O, 2h, 800 °C	20.50	0.41
O_2, 2h, 800 °C	20.48	0.48
